# BIG1 controls macrophage pro-inflammatory responses through ARF3-mediated PI(4,5)P2 synthesis

**DOI:** 10.1038/s41419-020-2590-1

**Published:** 2020-05-15

**Authors:** Lixin Liu, Sulin Zhang, Yirui Wang, Weilian Bao, Yile Zhou, Wenzhen Dang, Xu Wang, Haidong Li, Xinyue Cao, Yan You, Hao Fang, Xiaoyan Shen

**Affiliations:** 1 Department of Pharmacology & The Key Laboratory of Smart Drug Delivery, Ministry of Education, School of Pharmacy, Fudan University, Shanghai, China; 2 Department of Anesthesiology, Minhang Branch, Zhongshan Hospital, Fudan University, Shanghai, China; 3 Department of Anesthesiology, Zhongshan Hospital Affiliated Fudan University, Shanghai, China

**Keywords:** Acute inflammation, Sepsis

## Abstract

Sepsis is caused by a dysregulated host inflammatory response to serious infections resulting in life-threatening organ dysfunction. The high morbidity and mortality make sepsis still a major clinical problem. Here, we investigated the roles of Brefeldin A-inhibited guanine nucleotide-exchange factor 1 (BIG1) in the pathogenesis process of sepsis and the underlying mechanisms. We found myeloid cell-specific BIG1 knockout (BIG1 cKO) significantly reduced the mortality and organ damage in LPS-induced and CLP-induced polymicrobial sepsis mouse model. The serum concentration and mRNA expression of pro-inflammatory cytokines including TNF-α, IL-6, IL-1β, and IL-12 were obviously decreased in BIG1 cKO mice. In bone marrow-derived macrophages or THP-1 cells, BIG1 deficiency caused an inhibited ARF3 activation, which reduced PI(4,5)P2 synthesis and the recruitment of TIRAP to the plasma membrane through inhibiting the activation of PIP5K induced by LPS, and eventually resulted in the inhibitory activity of TLR4-MyD88 signaling pathway. These results reveal a crucial new role of BIG1 in regulating macrophage inflammation responses, and provide evidence for BIG1 as a potential promising therapeutic target in sepsis.

## Introduction

Sepsis is considered to be a life-threatening organ dysfunction caused by a dysregulated host response to infection^1,2^. Annually, global estimates of 31.5 million people are affected by sepsis with potentially up to 5.3 million deaths worldwide^3^. Despite significant advances in the early identification and treatment of sepsis have been achieved, the occurrence of sepsis-induced multiple organ failure and the resulting deaths are still a major clinical problem and clearly impose a substantial global burden^4,5^. So far, an effective treatment for sepsis is still lacking^6,7^. Thus, sepsis has been listed as a global health priority by adopting a resolution to improve the prevention, diagnosis, and management by the World Health Organization in 2017^8^.

Macrophages have comprehensive effects on immune homeostasis and inflammatory response, and play essential roles during the pathological process of sepsis. In the early stage of sepsis, macrophages are excessively activated through the binding of toll-like receptor (TLR) with pathogen-associated molecular patterns (PAMPs) of the invading pathogen, such as lipopolysaccharide (LPS) in gram-negative bacteria, resulting in the excessive secretion of massive amounts of pro-inflammatory cytokines such as tumor necrosis factor alpha (TNF-α), interleukin-1β (IL-1β), and interleukin-6 (IL-6)^9^, which is considered as one of the leading causes for the high mortality in the early stage of sepsis^10,11^. Therefore, controlling excessive inflammatory response of macrophages in the early stage of sepsis can be of great benefits for sepsis-caused mortality^12^.

Brefeldin A-inhibited guanine nucleotide-exchange factor 1 (BIG1) belongs to ADP ribosylation factor (ARF) guanine nucleotide exchange factor (GEF) of the Sec7 family. It catalyzes the activation of class I ARFs (ARF1 and ARF3) by accelerating replacement of bound GDP with GTP^13–16^ to initiate vesicle formation for transport between the Golgi and the plasma membrane, contributing to various cellular dynamics including cell spreading and adhesion, cell migration in wound healing, and neurite outgrowth^17–19^. Our recent experiments showed that the expression of BIG1 was regulated by LPS in macrophages. In the present study, we then explored the functions and the detailed mechanisms of BIG1 in the pathological process of sepsis.

## Materials and methods

### Myeloid cell lineage BIG1 conditional knockout mice generation and validation

Lyz2-Cre mice and BIG1 floxed mice (C57BL/6J background), 8 weeks old, were purchased from Shanghai Model Organisms Center, Inc. (Shanghai, China). Mice were maintained with free access to pellet food and water in plastic cages at 21 ± 2 °C and kept on a 12 h light/dark cycle. All animal procedures were performed following the “Guide for the Care and Use of Laboratory Animals” published by the National Institutes of Health (NIH) and were approved by the Ethics Committee of Experimental Research, Shanghai Medical College, Fudan University. Lyz2-Cre mice were mated with BIG1 floxed mice to generate Lyz2-cre^+^BIG1^fl/fl^ mice (referred to as BIG1 conditional knockout mice (cKO)) and Lyz2-cre^−^BIG1^fl/fl^ mice (referred to as WT mice).

Genotyping identification of BIG1^fl/fl^ mice was carried out by PCR using the following primers: 5′-aaggtttgtgtgatttcacttgttttat-3′ (sense) and 5′-gcctttatgctgaagtacacttttgaat-3′ (antisense). The floxed and the wild-type alleles displayed ~418 and ~255 bp, respectively. Genotyping identification of Lyz2-Cre mice was carried out by PCR using the following primers: 5′-agcgatggatttccgtctctgg-3′ (sense) and 5′-agcttgcatgatctccggtattgaa-3′ (antisense). Lyz2-Cre-positive mice displayed 272 bp, while Lyz2-Cre-negative mice had no band displayed. Myeloid cell lineage BIG1 conditional knockout mice were validated by simultaneous genotyping identification of BIG1^fl/fl^ and Lyz2-Cre.

### LPS-induced sepsis mouse model

To induce endotoxin shock, male BIG1-cKO mice and their WT littermate mice aged 8 to 10-weeks were administered with LPS at a dose of 50 mg/kg body weight by intra-peritoneal injection. The control group mice were administered with the same volume of normal saline.

### Cecal ligation and puncture (CLP)-induced sepsis mouse model

For CLP-induced sepsis model, male BIG1-cKO mice and their WT littermate mice aged 8 to 10-weeks were subjected to CLP as described previously^20,21^. Briefly, after general anesthesia, the cecum was ligated at 1.0 cm from the tip of the cecum, pierced twice with an 18-gauge needle, and gently squeezed to expel a small amount of fecal materials and then returned to the abdominal cavity. Sham-operated mice underwent the same laparotomy without CLP. The mice were given pre-warmed saline (0.1 ml/g body weight) subcutaneously after surgery.

### Antibodies and reagents

The following primary antibodies were used: mouse monoclonal anti-β-Actin (12262, 1:1000, Cell Signaling), mouse monoclonal anti-GAPDH (51332, 1:1000, Cell Signaling), rabbit polyclonal anti-BIG1 (A300-998A, 1:1000, Bethyl), rabbit monoclonal anti-IκBα (4814, 1:1000, Cell Signaling), rabbit monoclonal anti-pS536-p65 (3033, 1:1000, Cell Signaling), rabbit monoclonal anti-pS176/180-IKKα/β (2697, 1:1000, Cell Signaling), rabbit monoclonal anti-TLR4 (14358, 1:1000, Cell Signaling), rabbit monoclonal anti-MyD88 (4283, 1:1000, Cell Signaling), rabbit polyclonal anti-TIRAP (ab17218, 1:1000, Abcam), mouse monoclonal anti-PIP2 (sc53412, 1:200, Santa Cruz), rabbit polyclonal anti-ARF1 (PA1-127, 1:2000, Thermo Fisher), mouse monoclonal anti-ARF3 (610784, 1:500, BD Biosciences), mouse monoclonal anti-ARF5 (H00000381-M01, 1:500, Abnova), rabbit polyclonal anti-ARF6 (ab77581, 1:1000, Abcam), and mouse monoclonal anti-Myc-Tag (2276, 1:1000, Cell Signaling). Chemical reagents used are as follows: LPS (L4516, Sigma-Aldrich; L23351, Invitrogen), R848 (tlrl-r848-5, Invitrogen), CpG ODN (tlrl-2395-5, Invitrogen), FSL-1 (tlrl-fsl, Invitrogen), Pam3csk4 (tlrl-pms, Invitrogen).

### Cell lines

Human monocytic THP-1 cells were purchased from American Type Culture Collection (TIB-202, ATCC). THP-1 cells were cultured in RPMl 1640 supplemented with 10% FBS (04-001-1A-US, Biological Industries) and 0.05 mM β-mercaptoethanol (21985, Invitrogen). Mouse peritoneal macrophages were isolated from the WT and BIG1-cKO mice and cultured in DMEM (10569010, Gibco) with 10% FBS and 1% penicillin-streptomycin. Bone marrow-derived macrophages (BMDMs) were isolated as described previously^22,23^ and cultured in DMEM supplemented with 10% FBS and containing M-CSF (10 ng/ml) for 7 days. All cells were incubated at 37 °C under 5% (v/v) CO_2_ atmosphere.

### Small interfering RNA (SiRNA) and plasmid transfection

SiRNA and plasmids were transfected into cells by using Lipofectamine RNAi Max (13778150, Invitrogen) and Lipofectamine 3000 (L3000015, Invitrogen) respectively, following manufacturer’s instructions. Plasmids used are as follows: Myc-mARF1 (MR201636, Origene), HA-mARF1 (Q71L) (79410, Addgene), Myc-mARF3 (MR201629, Origene), HA-mARF3 (Q71L) (79415, Addgene). Primers sequences used are as follows: negative control (AAUUCUCCGAACGUGUCACGU); h-Si-BIG1 (GUCCAAAUGUCCUCGCAUA); h-Si-ARF1 (GACAAGCACUGUAAUUAUA); h-Si-ARF3 (GACUGUACAUGUUUAAUAA); m-Si-ARF1 (CAGAACACCCAAGGCUUGAUCUUCG); m-Si-ARF3 (CCGUGCUGUAUUGUCACAAGAACAU).

### Fluorescence microscopy

After treatment, BMDMs were fixed with 4% paraformaldehyde at room temperature (RT) for 15 min, then blocked for 30 min with 10% goat serum in PBS containing 0.1% saponin at RT. Cells were then incubated with primary antibodies for 2 h at RT, followed by incubation with Goat anti-mouse AlexaFluor 488 or Goat anti-rabbit AlexaFluor 594 antibodies (in blocking buffer) for 1 h. The coverslips were mounted in Prolong Gold anti-fade reagent with DAPI and then cells were visualized under confocal microscope (ZEISS LSM710, Germany).

### Flow cytometry analysis

BMDMs (2 × 10^5^) stimulated with LPS (100 ng/ml) for a certain time were blocked with anti-CD16/CD32 antibodies (Fc blocking) for 20 min on ice after being washed with PBS, and then stained with PE-labeled anti-mouse TLR4 for 30 min on ice. Then cells were washed twice with PBS to remove unbound antibody, followed by flow cytometry analysis (BD FACSAria II, America).

### Cell surface protein isolation

Pierce cell surface protein isolation kit was used to isolate cell surface proteins according to the manufacturer’s protocol. Cells on 100-mm plates were washed three times with ice-cold PBS, then incubated (30 min, 4 °C) in 10 ml of PBS containing 0.24 mg/ml EZ-link sulfo-NHS-SS-biotin for labeling surface protein. After 30 min, reaction-quenching solution was added. Cells were then washed twice with TBS, collected with 100 μl of lysis buffer. Cell lysates were mixed end over end (1 h, room temperature) with commensurable immobilized NeutrAvidin agarose beads. After washed with TBS, the biotinylated proteins were eluted in SDS sample buffer containing 50 mM DTT.

### Western blot

After treatment, proteins from cells were isolated by lysing in RIPA buffer on ice. BCA protein assay kit was used to determine the concentration of protein. Then equal amounts of proteins were separated by SDS-PAGE and transferred to nitrocellulose membranes. Membranes were blocked with 5% milk diluted with TBST and incubated with the specific primary antibodies overnight at 4 °C, followed by incubation with appropriate HRP-conjugated secondary antibodies for 1 h at RT. The immune complexes were imaged by chemiluminescence imaging system. The intensities of the blots were quantified by densitometry using Quantity One software (Bio-Rad) according to manufacturer’s instructions and a linear relationship between amount of loaded protein and density of the band was confirmed by Quantity One.

### RNA isolation and RT-qPCR

For RT-qPCR analysis, total RNA was extracted from cells by Trizol reagent. After reverse transcription, real-Time PCR was performed using SYBR in CFX96TM Real-Time quantitative PCR Detection System (Bio-Rad). The profile of thermal cycling consisted of initial denaturation at 95 °C for 30 s, 40 cycles at 95 °C for 5 s, and 60 °C for 30 s. All primers used for q-PCR analysis were synthesized by HuaGene (Shanghai, China). The specificity of each primer pair was confirmed by melting curve analysis and agarose-gel electrophoresis. β-Actin was used as an internal control. The primer sequences used in q-PCR are listed in Supplementary Table 1.

### Dot blot

After treatment, the whole cell lysates from BMDM cells were extracted. The lysates were spotted onto a nitrocellulose membrane, followed by blockage with 5% skim milk for 2 h at room temperature. Then, the membranes were incubated with PIP2 antibody (1:200, Santa Cruz) or GAPDH (1:1000, Cell Signaling) at 4 °C overnight. After incubation with a homologous HRP-conjugated secondary antibody at room temperature for 1 h, the membranes were detected using chemiluminescence imaging system with Clarity Western ECL Substrate.

### Immunoprecipitation

For immunoprecipitation analysis, transfected cells were lysed in lysis buffer (50 mM Tris-HCl, pH 7.5, 150 mM NaCl, 1% Nonidet P40, protease inhibitors and 0.1% SDS-Na) for 30 min and centrifuged at 13,000×*g* for 15 min. Cell lysates were incubated with 30 μl of anti-Myc agarose for 6 h at 4 °C. Immunocomplexes were washed three times with 1 ml of lysis buffer and then analyzed by western blot.

### Enzyme-linked immunosorbent assay (ELISA)

ELISA kits for tumor necrosis factor (TNF)-α (Cat#1217202), IL-6 (Cat#1210602), IL-1β (Cat#1210122) and IL-12 (Cat#1211232) were purchased from Dakewe Biotech Co. Ltd (Shenzhen, China). Levels of TNF-α, IL-6, IL-1β, and IL-12 in the cell culture medium and mouse serum were measured by ELISA according to manufacturer instructions.

### ARF reintroduction assay

Recombinant adenovirus vector (Ad-Vector served as a negative control) and adenovirus vector carrying ARF gene (Ad-ARF) were constructed by Genechem (Shanghai, China). For ARF reintroduction assay, BIG1 KO BMDMs were infected with Ad-ARF or Ad-Vector virus in the presence of 4 μg/ml of polybrene for 24 h.

### Quantification of ARF3 activity

To evaluate the activation profile of ARF3, we measured the levels of GTP-bound ARF3 by using the ARF-binding domain of GGA1 protein fused with GST, which binds the activated ARF3 (ARF3-GTP). WT and BIG1^−/−^ BMDMs were lysed on ice, and the ARF3-GTP was pulled down by GST-GGA1 and visualized by Western blot. The intensities of the ARF3-GTP blots were quantified by densitometry using Quantity One software.

### H&E staining

H&E staining assay was performed by Servicebio Inc. (Shanghai, China). Briefly, tissue samples were fixed in 4% paraformaldehyde and embedded in paraffin. Liver and lung tissues were sectioned (5 μm) for H&E staining and the stained sections were analyzed by a pathologist using a light microscope (Olympus, Tokyo, Japan).

### Quantification of PIP5K activity

The PIP5K activity was measured according to manufacturer instruction (Echelon Biosciences, K-5700). In briefly, WT and BIG1^−/−^ BMDMs treated with LPS (100 ng/ml) for 30 min, were lysed with sonication and freeze thaw cycles in the complete reaction buffer. The samples of cell lysates (10 μl) were added into 4 × PI(4)P solution (10 μl) per well, followed by adding 20 μl of the 2× ATP solution. The reaction was incubated at 37 °C for 2 h. After incubation, LATP detector (K-LUMa, 40 μl) was added into each well and incubated for at least 10 min at room temperature in the dark. Then, the luminescence was measured at 550 nm.

### Statistics

All the data were expressed as mean ± SEM. Statistical analysis was processed by GraphPad Prism version 6.0. Student’s *t*-test or one-way ANOVA was used to compare the mean values of the groups. Survival curves were calculated according to Kaplan–Meier method; survival analysis was performed using the logrank test. *P* < 0.05 was considered to be significant.

## Results

### BIG1 deficiency inhibited LPS-stimulated inflammatory response in BMDMs and THP-1 derived macrophages

**Fig. 1:**
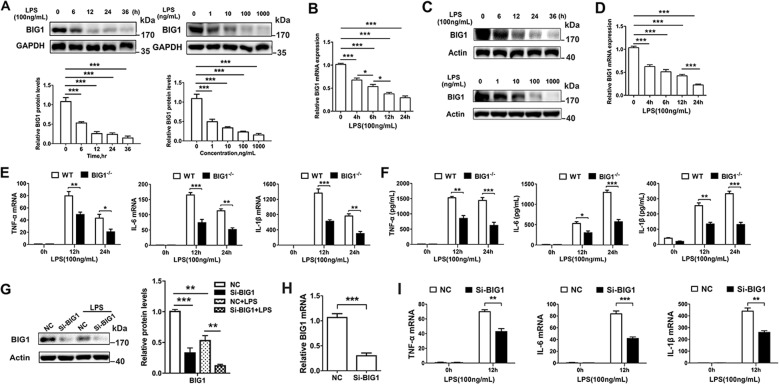
BIG1 deficiency inhibited LPS-stimulated inflammatory response in BMDMs and THP-1-derived macrophages. **a** BMDMs from WT mice were treated with 100 ng/ml LPS for 0, 6, 12, 24, and 36 h (left panel) or treated with different concentration of LPS (1, 10, 100, and 1000 ng/ml) for 12 h (right panel), the BIG1 protein levels were analyzed by Western blot, followed by the densitometry quantification of BIG1 (down panel). **b** BMDMs from WT mice were treated with LPS (100 ng/ml) for 0, 4, 6, 12, and 24 h and then BIG1 mRNA was evaluated by RT-qPCR. **c** THP-1 cells were treated with 100 ng/ml LPS for 0, 6, 12, 24, and 36 h (up panel) or treated with different concentration of LPS (1, 10, 100, and 1000 ng/ml) for 12 h (down panel) after incubating with PMA (100 ng/ml) for 24 h, the indicated protein levels were analyzed by Western blot. **d** Total RNA was extracted from THP-1 cells treated with LPS (100 ng/ml) for 0, 4, 6, 12, and 24 h after incubating with PMA (100 ng/ml) for 24 h, and the level of BIG1 mRNA was evaluated by RT-qPCR. **e**, **f** WT and BIG1^−/−^ BMDMs were treated with LPS (100 ng/ml) for the indicated time. Total RNA was extracted and the levels of TNF-α, IL-6, and IL-1β mRNA were measured by RT-qPCR (**e**); and the concentrations of TNF-α, IL-6, and IL-1β in the cell culture medium were measured by ELISA (**f**). **g** THP-1-derived macrophages transfected with negative control siRNA (NC) or BIG1 siRNA (Si-BIG1) were treated with LPS (100 ng/ml) for 12 h, the indicated protein levels were analyzed by Western blot (left panel) and quantified by densitometry (right panel). **h** THP-1-derived macrophages transfected with negative control siRNA (NC) or BIG1 siRNA (Si-BIG1), and BIG1 mRNA was measured by RT-qPCR to confirm the interference efficiency of BIG1 siRNA (Si-BIG1). **i** THP-1-derived macrophages transfected with negative control siRNA (NC) or BIG1 siRNA (Si-BIG1) were treated with LPS (100 ng/ml) for the indicated time, the levels of TNF-α, IL-6, IL-1β mRNA were measured by RT-qPCR. Data show pooled technical replicates from three independent experiments (**a, b, d**–**i**). All immunoblot data are representative of three independent experiments with similar results. Data are shown as mean ± SEM. *P < 0.05, **P < 0.01, ***P < 0.001 (Student’s *t*-test in **e**–**i**; one way ANOVA in **a**, **b**, **d**).

The role of BIG1 in inflammation is currently unclear. In order to explore the possible involvement of BIG1 in infective inflammation, we firstly detected whether the expression of BIG1 in bone marrow-derived macrophages (BMDMs) was changed after LPS stimulation. Interestingly, we found that the protein level of BIG1 was reduced by LPS stimulation in a time-dependent and dose-dependent manner (Fig. 1a). The results from RT-qPCR showed that the mRNA levels of BIG1 were also time-dependently downregulated by LPS (Fig. 1b), suggesting that the decreased BIG1 protein level was transcriptionally downregulated by LPS treatment. This phenomenon was also observed in human THP-1 derived macrophages (Fig. 1c, d). To further explored the impact of BIG1 downregulation in LPS-induced inflammatory response, we established Lyz2-Cre^+^BIG1^fl/fl^ (BIG1 cKO) and Lyz2-Cre^−^BIG1^fl/fl^ (WT) mice (Fig. S1a). In the BMDMs from BIG1 cKO mice, BIG1 mRNA and protein levels were almost abolished (Fig. S1b, c), suggesting that BIG1 cKO mice was successfully established. Then, we compared the levels of TNF-α, IL-6, and IL-1β in WT and BIG1^−/−^ BMDMs stimulated with LPS for 12 or 24 h. The results from RT-qPCR and ELISA showed that BIG1 deficiency inhibited both the mRNA expression and secretion of pro-inflammatory cytokine TNF-α, IL-6, and IL-1β (Fig. 1e, f). To confirm these results, THP-1-derived macrophages were subjected to BIG1 siRNA, and the interference efficiency was confirmed by Western blot and RT-qPCR. As shown in Fig. 1g, h, both protein level and mRNA of BIG1 were reduced by LPS treatment, and BIG1 siRNA significantly downregulated the expression of pro-inflammatory cytokine TNF-α, IL-6, and IL-1β mRNA (Fig. 1i). These results provided compelling evidence that BIG1 deficiency inhibited LPS-stimulated inflammatory response in BMDMs and THP-1-derived macrophages, suggesting that the downregulation of BIG1 by LPS may be a self-protective mechanism.

### BIG1 only responded to LPS stimulation in BMDMs and THP-1-derived macrophages

**Fig. 2:**
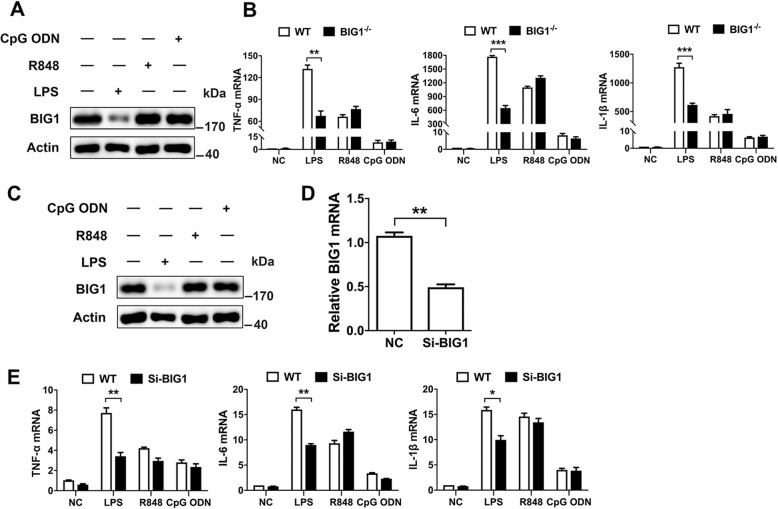
BIG1 only responded to LPS stimulation in BMDMs and THP-1-derived macrophages. **a** WT BMDMs were stimulated with LPS (100 ng/ml) for 12 h, R848 (10 nM) for 24 h, CpG ODN (5 µM) for 24 h, the indicated proteins were analyzed by Western blot. **b** WT and BIG1^−/−^ BMDMs were stimulated with LPS (100 ng/ml) for 12 h, R848 (10 nM) for 24 h, CpG ODN (5 µM) for 24 h. After treatment, the levels of TNF-α, IL-6, and IL-1β mRNA were measured by RT-qPCR. **c** THP-1 cells were treated with LPS (100 ng/ml) for 12 h, R848 (10 nM) for 24 h, CpG ODN (5 µM) for 24 h after incubating with PMA (100 ng/ml) for 24 h, the indicated protein were analyzed by Western blot. **d**, **e** THP-1-derived macrophages transfected with negative control siRNA (NC) or BIG1 siRNA (SiRNA) were untreated or treated with LPS (100 ng/ml) for 12 h, R848 (10 nM) for 24 h, CpG ODN (5 µM) for 24 h. Total RNA was extracted. The levels of BIG1, TNF-α, IL-6, and IL-1β mRNA were measured by RT-qPCR. Data show pooled technical replicates from three independent experiments (**b**, **d**, **e**). All immunoblot data are representative of three independent experiments with similar results. Data are shown as mean ± SEM. *P < 0.05, **P < 0.01, ***P < 0.001 (Student’s *t*-test in **b**, **d**, **e**).

In macrophages, the main receptor for LPS is TLR4. To explore whether the other TLRs were also involved in BIG1 signaling, BMDMs and THP-1-derived macrophages were stimulated with LPS, TLR7 agonist R848, and TLR9 agonist CpG ODN, respectively. As shown in Fig. 2a, b, the protein level of BIG1 was decreased in response to LPS, but not R848 and CpG ODN stimulation in BMDMs; and BIG1 deficiency only inhibited LPS, but not R848 and CpG ODN-induced TNF-α, IL-6, and IL-1β mRNA expression. Similarly, BIG1 was reduced in THP-1-derived macrophages treated with LPS, but not R848 and CpG ODN (Fig. 2c), and BIG1 interference also resulted in an inhibition of TNF-α, IL-6, and IL-1β mRNA expression in THP-1-derived macrophages (Fig. 2d, e). To explore whether TLR2 was also involved in BIG1 signaling, BMDMs and THP-1-derived macrophages were stimulated with both FSL-1 and Pam3csk4. As shown in Fig. S2a, b, the protein level of BIG1 has no change, and BIG1 deficiency did not inhibited FSL-1-induced and Pam3csk4-induced TNF-α, IL-6, and IL-1β mRNA expression. Similarly, BIG1 was not reduced in THP-1-derived macrophages treated with FSL-1 and Pam3csk4 (Fig. S2c), and BIG1 interference also did not affect the expression of TNF-α, IL-6, and IL-1β mRNA in THP-1-derived macrophages (Fig. S2d). These results suggest that the downregulation of BIG1 induced by LPS mainly is related to TLR4 signaling in BMDMs and THP-1-derived macrophages.

### Myeloid cell-specific BIG1 KO (BIG1 cKO) protected mice from LPS-induced endotoxemic shock

**Fig. 3:**
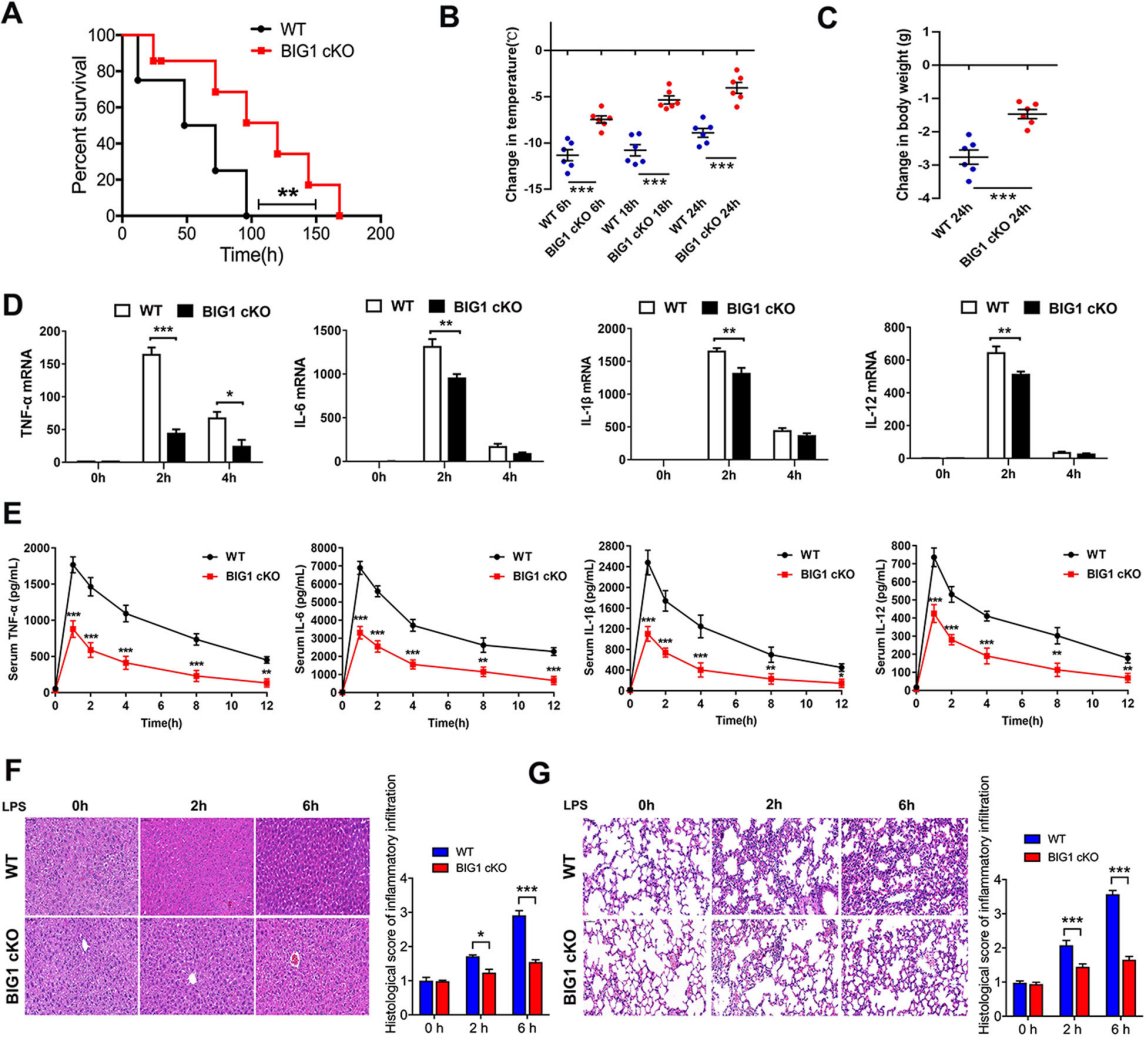
Myeloid cell-specific BIG1 KO protected mice from LPS-induced endotoxemic shock. WT and myeloid cell-specific BIG1 KO (BIG1 cKO) mice were intra-peritoneally administered with either LPS (50 mg/kg) or isometric saline. **a** Survival curves of LPS-treated WT and BIG1 cKO mice were calculated according to the Kaplan–Meier method. Survival analysis was performed using log-rank test (**P < 0.01; *n* = 10). **b** Body temperature of WT and BIG1 cKO mice were measured at the indicated time period. Mean ± SEM is presented and analyzed by Student’s *t*-test (***P < 0.001; *n* = 6). **c** Body weight loss of WT and BIG1 cKO mice were measured at the indicated time period. Mean ± SEM is presented and analyzed by Student’s *t*-test (***P < 0.001; *n* = 6). **d** The mRNA levels of the indicated cytokines in peritoneal macrophages from WT and BIG1 cKO mice injected intra-peritoneally with LPS (50 mg/kg) were measured by RT-qPCR at the indicated time period. Mean ± SEM is presented and analyzed by Student’s *t*-test (*P < 0.05, **P < 0.01, ***P < 0.001; *n* = 6). **e** Blood was collected from WT and BIG1-cKO mice injected intra-peritoneally with LPS (50 mg/kg) at the indicated time points. Serum levels of the indicated cytokines from WT and BIG1 cKO mice were measured by ELISA at the indicated time point. Mean ± SEM is presented and analyzed by Student’s *t*-test (*P < 0.05, **P < 0.01, ***P < 0.001; *n* = 6). **f**, **g** Liver and lung sections from WT and BIG1 cKO mice were subjected to H&E staining at the indicated time point. The representative images were shown (original magnification, ×400;Scale bars, 50 μm). The inflammatory infiltration score was quantified. Data are presented as mean ± SEM. *P < 0.05, **P < 0.01, ***P < 0.001; *n* = 6; Student’s *t*-test.

Macrophages have comprehensive effects on immune homeostasis and inflammatory response, and play essential roles during the pathological process of sepsis. The above data in macrophages suggest that BIG1 was involved in the regulation of LPS-stimulated inflammatory response. Therefore, we speculated that BIG1 deficiency may ameliorate pathological changes of sepsis. Therefore, we firstly examined the survival rates of WT and BIG1 cKO mice following LPS injection. As shown in Fig. 3a, LPS injection induced severe sepsis and resulted in a mortality rate of up to 80% within 48 h in WT mice (80% on 48 h, 100% on 96 h). Compared with septic WT mice, BIG1 cKO mice treated with LPS had a significantly reduced mortality rate (50% on 48 h, 80% on 96 h). There was no mortality in the groups of mice received saline injection. Consistently, BIG1 cKO mice exhibited lower clinical signs of sepsis than WT mice, including hypothermia (Fig. 3b) and weight loss (Fig. 3c). In order to assess the difference of inflammatory response between WT and BIG1 cKO septic mice, we isolated the peritoneal macrophages and serum after intraperitoneal injection of LPS. As shown in Fig. 3d, e, the mRNA level in the peritoneal macrophages and serum concentration of pro-inflammatory cytokines including TNF-α, IL-6, IL-1β, and IL-12 were increased in LPS-treated WT mice, but reduced in BIG1 cKO mice. To further estimating the organ damage, serum concentration of ALT and aspartate aminotransferase (AST), and the mRNA level of pro-inflammatory cytokines in liver and lung tissue were detected (Fig. S3a–d). Compared with WT septic mice, myeloid cell-specific BIG1 cKO mice had a significant decrease in serum ALT and AST, as well as the mRNA expression of pro-inflammatory cytokines in liver and lung tissue. Furthermore, the inflammatory injury was evaluated by HE staining. Livers and lungs from LPS-induced BIG1 cKO mice showed alleviative inflammation and tissue injury compared with those from LPS-induced WT mice (Fig. 3f, g). Overall, these data suggest that BIG1-mediated inflammatory response may play a crucial role in LPS-induced sepsis and contribute to the high mortality of sepsis.

### BIG1-cKO mice were protected from polymicrobial sepsis in the CLP model

**Fig. 4:**
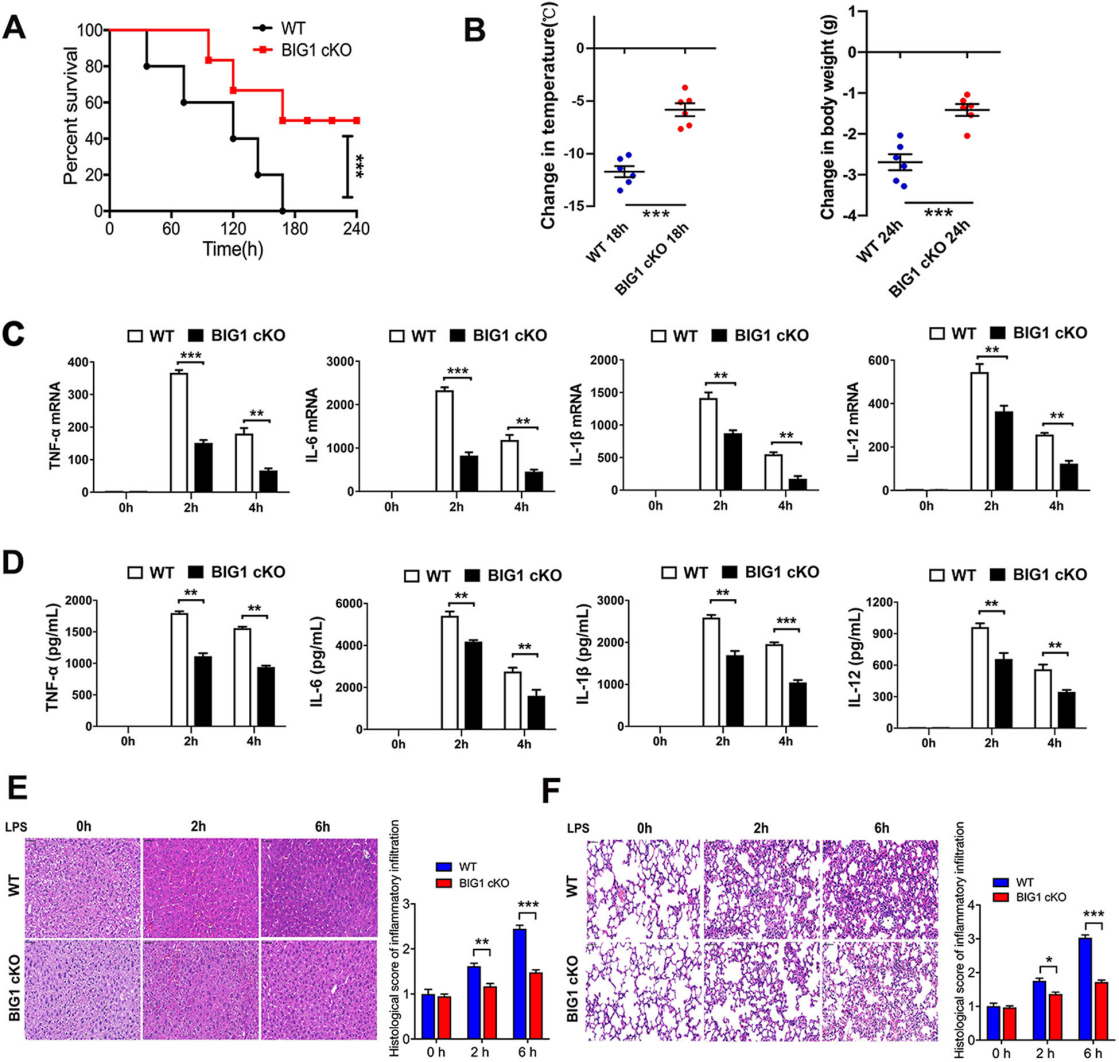
BIG1-cKO mice were protected from polymicrobial sepsis in the CLP model. WT and BIG1-cKO mice were subject to CLP surgery. **a** The mortality of WT and BIG1-cKO septic mice were assessed daily for up to 10 days. Survival rate were calculated according to the Kaplan–Meier method. Survival analysis was performed using log-rank test (***P < 0.001, *n* = 10). **b** Loss of body temperature and weight in WT mice and their littermates BIG1-cKO mice were measured at the indicated time point. Mean ± SEM is presented and analyzed by Student’s *t*-test (***P < 0.001; *n* = 6). **c** The mRNA levels of cytokines in peritoneal macrophages from CLP-treated WT and BIG1-cKO mice were measured at the indicated time point. Mean ± SEM is presented and analyzed by Student’s *t*-test (**P < 0.01, ***P < 0.001; *n* = 6). **d** Serum levels of TNF-α, IL-6, IL-1β, and IL-12 from WT and BIG1 cKO mice were measured by ELISA at the indicated time point. Mean ± SEM is presented and analyzed by Student’s *t*-test (*P < 0.05, **P < 0.01, ***P < 0.001; *n* = 6). **e**, **f** Liver and lung sections from WT and BIG1 cKO mice were subjected to H&E staining at the indicated time point. The representative images were shown (original magnification, ×400; Scale bars, 50 μm). The inflammatory infiltration score was quantified. Data are presented as mean ± SEM. *P < 0.05, **P < 0.01, ***P < 0.001; *n* = 6; Student’s *t*-test.

To further investigate the role of BIG1 in sepsis, the bacterial endotoxemia model was used. As illustrated in Fig. 4a, polymicrobial peritonitis after CLP induced severe sepsis that resulted in a mortality rate of up to 70% within 96 h in WT mice (70% on 96 h, 100% on 168 h). While, BIG1 cKO mice with polymicrobial sepsis had a significant reduction in the mortality rate (30% on 96 h, 70% on 168 h). There was no mortality in the groups of mice subjected to sham operation. We then compared the changes of temperature and body weight between BIG1 cKO and WT septic mice. As shown in Fig. 4b, compared with WT septic mice, BIG1 cKO septic mice had an alleviative hypothermia and weight loss. In order to test inflammatory factors in septic mice, we isolated the peritoneal macrophages at 2 and 4 h after CLP-induced sepsis. The levels of inflammatory cytokines were detected respectively using RT-qPCR. As shown in Fig. 4c, similar to LPS model, CLP-induced a marked increase in mRNA levels of pro-inflammatory cytokines including TNF-α, IL-6, IL-1β, and IL-12 in WT mice. While, BIG1 cKO septic mice had much lower levels of TNF-α, IL-6, IL-1β, and IL-12 mRNA. Similar changes were also observed in the serum concentration of these cytokines (Fig. 4d). Organ damages were then estimated. As shown in Fig. S4a–d, compared with WT septic mice, myeloid cell-specific BIG1 cKO mice had significantly decreased serum ALT and AST, as well as the mRNA levels of pro-inflammatory cytokines in liver and lung tissue. HE staining on liver and lung tissue section revealed that BIG1 cKO significant alleviative inflammatory cell infiltration and tissue injury compared with WT septic mice (Fig. 4e, f). These data confirm that BIG1 plays a predominant role in mediating inflammatory response during CLP-induced polymicrobial sepsis and contributes to its high mortality.

### BIG1-mediated ARF3 activation was essential for LPS-stimulated inflammatory responses

**Fig. 5:**
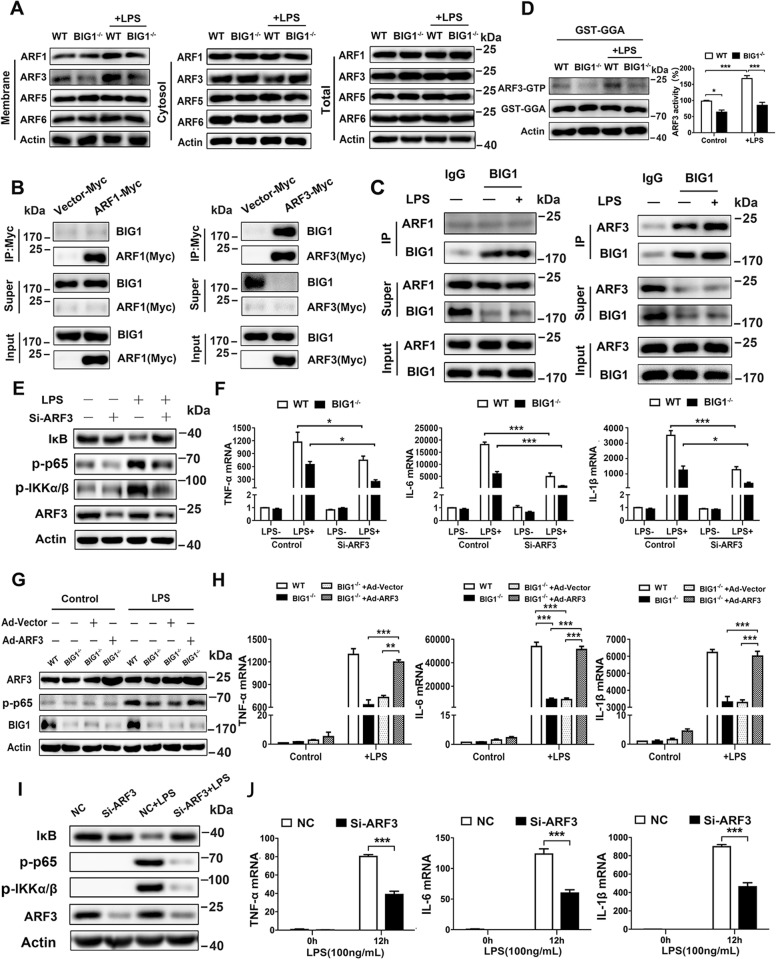
BIG1 mediated-ARF3 activation was essential for LPS-stimulated inflammatory response. **a** WT and BIG1^−/−^ BMDMs were stimulated with LPS (100 ng/ml) for 30 min, the total, membrane-bounded and cytosolic proteins were measured by Western blot with the indicated antibodies. **b** Cell lysates from WT BMDMs transfected with Myc-ARF1 or Myc-ARF3 were subjected to pull-down assay, followed by western blot with indicated antibodies. **c** Cell lysates from WT BMDMs with or without LPS (100 ng/ml) treatment for 30 min were subjected to immunoprecipitation with BIG1 antibody, followed by Western blot with the indicated antibodies. **d** WT and BIG1^−/−^ BMDMs were treated with LPS (100 ng/ml) for 30 min, the activated ARF3 (ARF3-GTP) was pulled down by GST-GGA and visualized by Western blot. The densitometry of ARF3-GTP was quantified by Quantity One software and the activity of ARF3 in WT and BIG1^−/−^ BMDMs was measured. **e** WT BMDMs transfected with Si-ARF3 or negative control siRNA were treated with or without LPS (100 ng/ml) for 30 min. Total lysates were subjected to Western blot with indicated antibodies. **f** WT and BIG1^−/−^ BMDMs were transfected with Si-ARF3 or negative control siRNA for 48 h before treated with LPS for 6 h. The mRNA levels of TNF-α, IL-6, and IL-1β were measured by RT-qPCR. **g**, **h** BIG1^−/−^ BMDMs transfected with active mutant ARF3 (Q71L) or vector were treated with or without LPS (100 ng/ml). Total lysates were subjected to Western blot after treatment for 30 min (**g**), and the mRNA levels of TNF-α, IL-6, and IL-1β were measured by RT-qPCR after treatment for 6 h (**h**). **i**, THP-1-derived macrophages transfected with negative control siRNA (NC) or si-ARF3 were treated with or without LPS (100 ng/ml) for 30 min. The cell lysates were subjected to immunoblotting with the indicated antibodies. **j** THP-1-derived macrophages transfected with negative control siRNA or si-ARF3 were treated without or with LPS (100 ng/ml) for 6 h. The expression of TNF-α, IL-6, and IL-1β mRNA were measured by RT-qPCR. Data show pooled technical replicates from three independent experiments (**d**, **f**, **h**, **j**). All immunoblot data are representative of three independent experiments with similar results. Data are shown as mean ± SEM. *P < 0.05, **P < 0.01, ***P < 0.001 (Student’s *t*-test in **f** and **j**; one way ANOVA in **d**, **h**).

BIG1, as a member of the ADP ribosylation factor (ARF) guanine nucleotide exchange factor (GEF) of the Sec7 family, catalyzes the activation of class I ARFs (ARF1 and ARF3) by accelerating replacement of bound GDP with GTP to initiate the downstream membrane trafficking events^16,18,19,24–26^. To further find out which ARF was involved in BIG1-mediated inflammatory response, a series of screening and rescue experiments were conducted. Noticeably, BIG1 deficiency specifically caused a decrease in the membrane-bound ARF3 (Fig. 5a), and BIG1 was only pulled down by Myc-tagged ARF3 rather than Myc-tagged ARF1, indicating an interaction of ARF3 and BIG1 (Fig. 5b). Reciprocal immunoprecipitation by anti-BIG1 antibody showed that endogenous BIG1 also could precipitate endogenous ARF3, and LPS treatment enhanced the interaction of BIG1 and ARF3 (Fig. 5c). To confirm this result, ARF3 activity was detected. Compared with WT BMDMs, the ARF3-GTP level was steadily decreased in BIG1^−/−^ BMDMs, which suggest that the activity of ARF3 in BIG1^−/−^ BMDMs was indeed decreased (Fig. 5d). ARF3, but not ARF1 interference could mimic the inhibition of BIG1 deficiency in LPS-induced inflammatory response (Fig. 5e, f; Fig. S5a, b). To further confirm the role of ARF3 in BIG1-mediated inflammatory response, the rescue experiment was conducted. Active mutant ARF1 (Q71L) and active mutant ARF3 (Q71L) and empty vectors were transfected into BIG1^−/−^ BMDMs, respectively. As shown in Fig. 5g, h, and Fig. S5c, d, active ARF3, but not active ARF1 transfection reversed BIG1 KO-induced inhibition of NF-κB signaling pathway activation and pro-inflammatory gene expression. Next, ARF3 was interfered to verify its impact on human THP-1-derived macrophages. As shown in Fig. 5i, j, the activation of NF-κB signaling pathway was inhibited, and the transcription of the pro-inflammatory gene expression was also reduced in ARF3 depleted THP-1-derived macrophages. While, there is no significant difference between WT and ARF1 depleted THP-1-derived macrophages (Fig. S5e). These results suggest that BIG1-mediated ARF3 activation is essential for LPS-stimulated inflammatory response.

### Decrease of BIG1-mediated ARF3 activation reduced PIP2 production and prohibited the binding of TIRAP to the plasma membrane of macrophages induced by LPS

**Fig. 6:**
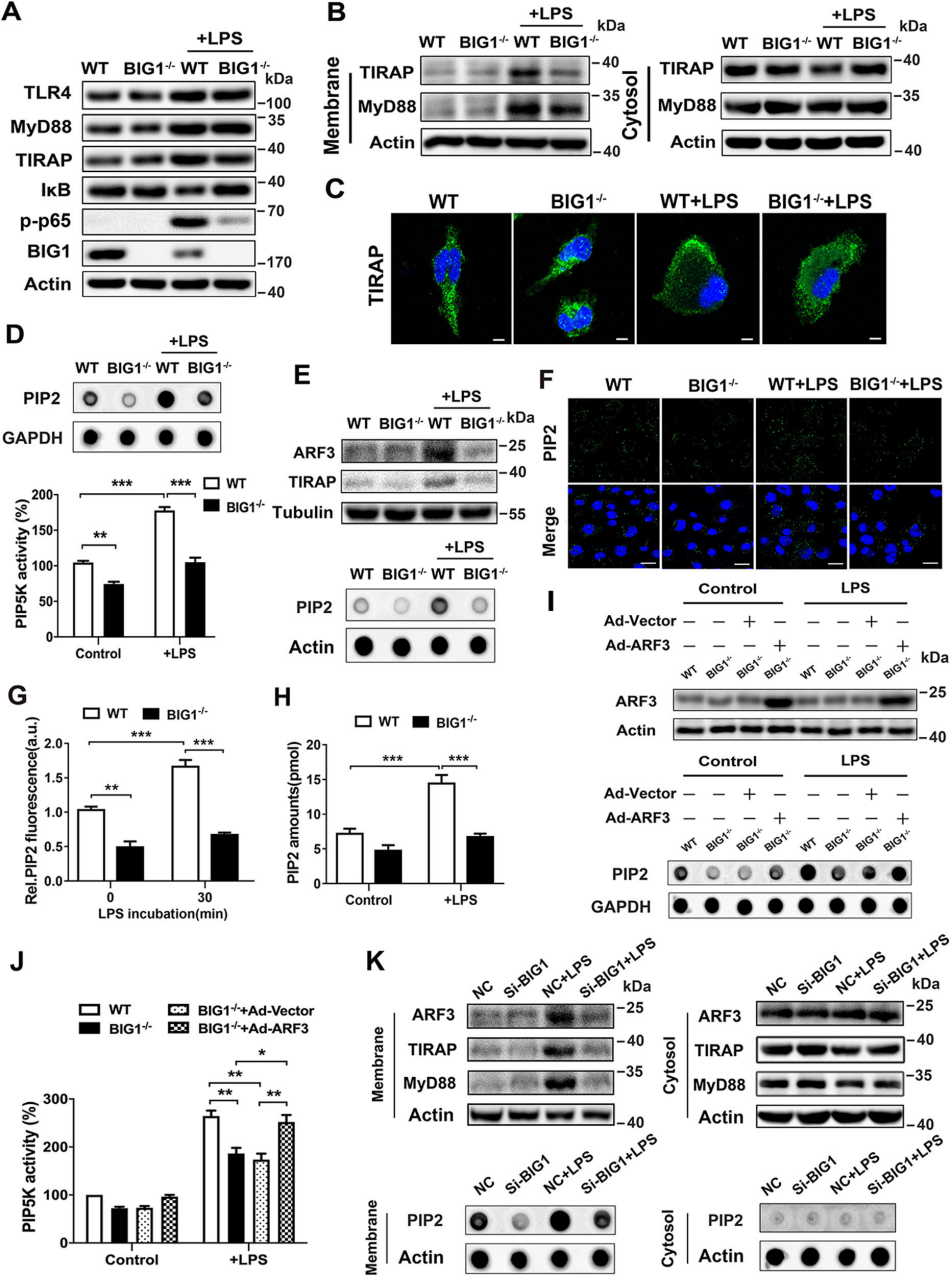
BIG1 deficiency reduced PIP2 production and prohibited the binding of TIRAP to the plasma membrane of macrophages induced by LPS. **a** WT and BIG1^−/−^ BMDMs were treated with or without LPS (100 ng/ml) for 30 min. The cell lysates were subjected to immunoblotting with the indicated antibodies. **b** The membrane-bounded and cytosolic proteins from WT and BIG1^−/−^ BMDMs stimulated with LPS (100 ng/ml) for 30 min were measured by immunoblotting with the indicated antibodies. **c** WT and BIG1^−/−^ BMDMs were treated with or without LPS (100 ng/ml) for 30 min, followed by confocal imaging analysis with TIRAP antibody. Scale bars, 20 μm. **d** WT and BIG1^−/−^ BMDMs were treated with LPS (100 ng/ml) for 30 min, followed by dot blot with the indicated antibodies (up panel). The activity of PIP5K was measured by luminescence assay kit (down panel). **e** The cell surface proteins from WT and BIG1^−/−^ BMDMs treated with LPS (100 ng/ml) for 30 min, were measured by dot blot and immunoblotting with the indicated antibodies, respectively. **f** WT and BIG1^−/−^ BMDMs were treated with or without LPS (100 ng/ml) for 30 min, followed by confocal imaging analysis with PIP2 antibody. Scale bars, 20 μm. **g** PIP2 fluorescent intensities from more than 50 individual cells were quantified in **f**. **h** WT and BIG1^−/−^ BMDMs were treated with or without LPS (100 ng/ml) for 30 min, then cellular PIP2 levels from extracted acidic lipids were measured by using the PIP2 mass ELISA kit. **i**, **j** BIG1^−/−^ BMDMs transfected with active mutant ARF3 (Q71L) or vector were treated with or without LPS (100 ng/ml) for 30 min, followed by dot blot and Western blot with the indicated antibodies (**i**) or measuring the activity of PIP5K (**j**). **k** THP-1-derived macrophages transfected with negative control siRNA or si-BIG1 were treated with or without LPS (100 ng/ml) for 30 min. The membrane-bounded and cytosolic proteins were isolated, followed by dot blotting with the indicated antibodies. Data show pooled technical replicates from three independent experiments (**d**, **g**, **h**, **j**). All immunoblot data are representative of three independent experiments with similar results. Data are shown as mean ± SEM. *P < 0.05, **P < 0.01, ***P < 0.001 (one way ANOVA in **d**, **g**, **h**, **j**).

To determine the mechanisms underlying BIG1 deficiency-mediated inhibition of LPS-induced inflammatory response, we examined the membrane-binding activation of TIRAP, which is the essential initial step in LPS-stimulated inflammatory signaling pathways. As shown in Fig. 6a, in WT BMDMs, LPS stimulation strongly induced IκB degradation and the phosphorylation of NF-κB in the total cell lysates, but the total TLR4, TIRAP, and Myd88 had no changes. While, BIG1 KO significantly inhibited IκB degradation and NF-κB phosphorylation (Fig. 6a). Next, we compared internalization of TLR4 induced by LPS between BIG1^−/−^ and WT BMDMs by flow cytometry and immunofluorescent staining, respectively, the data suggested that there is no significant difference (Fig. S6a–c). We also examined whether BIG1 deficiency had an effect on TLR2. We stimulated WT and BIG1^−/−^ BMDMs with TLR2 agonist BLP, the results showed that the protein levels of TLR2 had no significant difference between WT and BIG1^−/−^ BMDMs (Fig. S6d). We then isolated the membrane proteins and cytosolic proteins to detect the activation of MyD88-dependent pathways at 0.5 h of LPS stimulation. As shown in Fig. 6b, compared with WT BMDMs, the membrane-bounded TIRAP and MyD88 in BIG1^−/−^ BMDMs were decreased. Besides, the result of immunofluorescence staining also showed that membrane-bounded TIRAP was decreased in BIG1^−/−^ BMDMs after LPS stimulation, which were consistent with Western blots (Fig. 6c). This raised an important question how BIG1 deficiency caused a decrease in membrane localization of TIRAP. It has been reported that TIRAP contains a phosphatidylinositol 4,5-bisphosphate (PIP2) binding domain, which recruits TIRAP to the plasma membrane^27^, and then facilitates the bind of MyD88 to activate TLR4 to initiate signal transduction^27^. PIP5K was reported to be a direct effector of ARF1, and ARF1 active mutant selectively activated PIP5K, but not PLD, contributed to the increase of PIP2 synthesis^28^. Therefore, we hypothesized that the decrease of ARF3 activity caused by BIG1 deficiency may also inhibit PIP5K-mediated PIP2 production. To verify it, we examined the activity of PIP5K, and the production of PIP2 in WT and BIG1^−/−^ BMDMs. We found that BIG1 deficiency reduce the activity of PIP5K, as well as the production of PIP2 (Fig. 6d). Particularly the levels of PIP2, TIRAP, ARF3 associated with the cell plasma membrane were all reduced (Fig. 6e). Immunofluorescence confirmed the reduction of membrane location of PIP2 in BIG1^−/−^ BMDMs (Fig. 6f, g). The results from ELISA also showed the cellular PIP2 levels from extracted acidic lipids were reduced in the absence of BIG1 (Fig. 6h).

To further confirm the downregulation of PIP2 protein levels and PIP5K activity in BIG1^−/−^ BMDMs was due to the reduction of ARF3 activity, the over-expression of active mutant ARF3 was conduct in BIG1^−/−^ BMDMs. As shown in Fig. 6i, j, PIP2 levels and PIP5K activity in BIG1^−/−^ BMDMs transfected with active mutant ARF3 were significantly restored. To further confirm this phenotype, BIG1 was interfered to verify its impact on human THP-1-derived macrophages. The membrane and cytosolic proteins were obtained from THP-1-derived macrophages with or without LPS treatment, followed by dot blot or Western blot with the indicated antibodies. As shown in Fig. 6k, the levels of ARF3, PIP2, TIRAP, MyD88 associated with membrane were reduced in BIG1 knockdown THP-1-derived macrophages. These results suggest that the decrease of ARF3 activation by BIG1 KO reduces PIP2 production and prohibits the binding of TIRAP to the plasma membrane of macrophages induced by LPS, contributing to the improvement of sepsis in BIG1 cKO mice.

## Discussion

BIG1 mainly located in the Golgi apparatus, and functions in membrane trafficking between Golgi and plasma membrane which is medicated by activating first class ARFs through its Sec7 domain^26,29^. BIG1 also contains A-kinase anchoring protein (AKAP) sequences that can work as a scaffold protein for multimolecular assemblies to facilitate and limit cAMP signaling and effects of its other actions in space and time^30^. Recent studies have shown that BIG1 was involved in cell dynamics, including cell proliferation, adhesion, and migration, as well as the neurite outgrowth^17–19^. The requirement of BIG1 for tumor necrosis factor receptor-associated factor 2 to be recruited into TNF receptor 1 signaling complexes had been reported^31^, suggesting an new mode of action for BIG1 in cell surface receptor activation. Here, we report that BIG1 was required for TIRAP recruitment to the plasma membrane to assemble the myddosome for TLR4-MyD88-NF-κB signaling activation through ARF3 activation in macrophages, illuminating a new function of BIG1 in regulating macrophage function.

In sepsis, an invading pathogen initiates the immune response which is fails to return to homeostasis, and eventually lead to a complex biphasic syndrome characterized by sustained excessive inflammation and immune suppression^32^. At the early stage of sepsis, activated macrophages release a large amount of pro-inflammatory cytokines such as TNF-α, IL-1β, IL-6 to resolve invading pathogens and injured tissues, playing pivotal role in host defense. While, excessive pro-inflammatory cytokines produced by over-activated macrophages result in cardiovascular collapse and multiple organ dysfunction, becoming one of the leading causes of high mortality in early sepsis^10^. The uncontrollable macrophage-mediated pro-inflammatory responses and the consequent formation of cytokine storms ultimately lead to immunosuppression, which predisposes the patients survived early sepsis to long-term risk for life-threatening secondary infections, contributing to the high mortality of sepsis^33^. Therefore, controlling macrophages in an appropriate active state is essential for efficiently mounting a protective and balanced response to infections in sepsis in order to eliminate the pathogen and prevent tissue damage. In the present study, we report BIG1 is a new player in mediating the activation of macrophages. The expression of BIG1 in macrophages was regulated by LPS stimulation and the deficiency of BIG1 in myeloid cells inhibited macrophage-mediated pro-inflammatory responses and tissue damages in septic mice, suggesting that the downregulation of BIG1 by LPS may be a self-protective mechanism in sepsis.

The activation of macrophages is mediated by the binding of PAMPs (such as LPS) with pattern recognition receptors, in which the TLR are one of the most important pattern recognition receptor families. TLR4, as a receptor for LPS, is the dominating receptor involving in pro-inflammatory responses induced by Gram-negative bacillus^34^. In response to LPS stimulation, TLR4-LPS complex transmits a signal from the cytoplasmic region of TLR4 by recruiting a TIR domain-containing adapter protein (TIRAP) to the proximal lipid-like microdomain of the membrane^27,35^. TIRAP subsequently recruit a protein complex called myddosome that contains MyD88, the IRAK family of protein kinases and another TIR domain-containing adapter protein, resulting in the activation of transcription factor NF-κB and expression of a range of inflammatory cytokines^36^. TLR4 internalization is considered to be a mechanism to prevent uncontrolled inflammation and septic shock^37^. Our previous studies showed that BIG1 plays an important roles in regulating plasma membrane protein ABCA1 and GABAARs trafficking^38,39^. In spite of the well-known function of BIG1 in initiating vesicle formation for transport between the Golgi and the plasma membrane^17–19^, we did not observe the predicted impact of BIG1 deficiency on TLR4 internalization and trafficking, suggesting an unknown new function of BIG1 in TLR4-MyD88 signaling pathway.

It has been reported that the recognition of LPS by CD14 activates type I PI 4-phosphate 5-kinase (PIP5K) isoforms, resulting in the production of phosphatidylinositol 4, 5-bisphosphate (PIP2) in the plasma membrane. TIRAP contains a PIP2 binding domain through which TIRAP is recruited to the plasma membrane. The membrane-bound TIRAP then facilitates the delivery of MyD88 to the activated TLR4 for the initiation of signal transduction^27,40^. PIP2 is relatively enriched in the plasma membrane and maintained at a low level under resting conditions, while is upregulated by multifarious extracellular signals such as LPS^41^. Studies showed that PIP2 level is essential for many physiologic processes involved in cell signaling, actin dynamics, and vesicle trafficking^42–44^. It has been reported that the synthesis of PIP2 by PIP5K isoforms is tightly regulated by small GTPases^45^. Activation of PIP5K by ARF proteins, especially ARF1 and ARF6, has been well established^46,47^. ARF1 can directly activate PIP5K to increase PIP2 synthesis^28^; while ARF6 could organize PIP2 turnover at the plasma membrane through a direct interaction with PIP5K^48^, and facilitate TIRAP translocate to lipid rafts to mediate the activation of TLR4-MyD88-dependent NF-κB signaling^49–51^. In the present study, we found that BIG1 deficient macrophages had less plasma membrane-bounded TIRAP and Myd88, which was caused by the decreased production of PIP2 due to inactivation of ARF3. These changes could be reversed by ARF3 overexpression, suggesting that a novel function of BIG1-mediated ARF3 activation in regulating TLR4-MyD88 signaling pathway through PIP5K-mediated PIP2 synthesis.

**Fig. 7:**
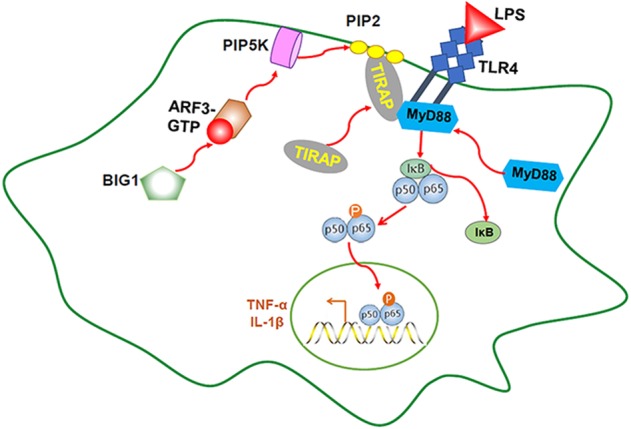
Schematic representation of BIG1 in sepsis. BIG1 plays a crucial role in mediating the activation of TLR4-MyD88 signaling pathways through its interaction with ARF3 during the pathological process of sepsis. BIG1 deficiency caused an inhibitory ARF3 activation, which inhibited the activation of PIP5K induced by LPS, resulting in the reduced PIP2 production and the recruitment of TIRAP to the plasma membrane. This caused an inhibitory activation of TLR4-MyD88 signaling pathway, and eventually decreased the production of pro-inflammatory cytokines, and alleviated the inflammatory injury resulted from sepsis.

In conclusion, this study showed that BIG1 plays a crucial role in mediating the activation of TLR4-MyD88 signaling pathways through its interaction with ARF3 during the pathological process of sepsis. BIG1 deficiency caused an inhibitory ARF3 activation, which reduced PIP2 production and the recruitment of TIRAP to the plasma membrane through inhibiting the activation of PIP5K induced by LPS, and eventually resulted in the inhibitory activity of TLR4-MyD88 signaling pathway. This is favorable the decrease of septic mortality by reducing the production of pro-inflammatory cytokines and inflammatory organ injury (Fig. 7). Our results disclose a crucial new role of BIG1 in regulating inflammation. Clinic confirmation in human sepsis is needed in favor of BIG1 as a potential promising therapeutic target for sepsis treatment.

## Supplementary information

Supplementary Table 1

Supplemental Figure Legends

Figure S1

Figure S2

Figure S3

Figure S4

Figure S5

Figure S6
