# Supplementary material for: BIG1 controls macrophage pro-inflammatory responses through ARF3-mediated PI(4,5)P2 synthesis
Source: Cell Death Dis. 2020 May 15;11(5):374. doi: 10.1038/s41419-020-2590-1 (PMC7229175; doi:10.1038/s41419-020-2590-1)
Supplement: Supplementary file 1 — Supplementary Table 1 [file 41419_2020_2590_MOESM1_ESM.docx]

**Supplementary Table 1. Real-time PCR Primer Sequences**

| **Gene** | **Forward (5’-3’)** | **Reverse (5’-3’)** |
| --- | --- | --- |
| mβ-Actin | CTGTCCCTGTATGCCTCTG | ATGTCACGCACGATTTCC |
| mBIG1 | ACGTGTAACTGCACCCTGG | TGATGGAGATGGGGGTTCG |
| mTNF-α | AGGCTGCCCCGACTACGT | GACTTTCTCCTGGTATGAGATAGCAAA |
| mIL-6 | ACAAGTCGGAGGCTTAATTACACAT | TTGCCATTGCACAACTCTTTTC |
| mIL-1β | TCGCTCAGGGTCACAAGAAA | CATCAGAGGCAAGGAGGAAAA |
| mIL-12 | ACAGCACCAGCTTCTTCATCAG | TCTTCAAAGGCTTCATCTGCAA |
| mARF1 | GCCTGAGGTCTATGCTCAGT | CCAAAGACCTGGCTTCCTGG |
| mARF3 | CCAGCGGACCATCTTCACC | GTATGGAGGACTTGACCCTGT |
| hβ-Actin  hBIG1  hARF1  hARF3  hTNF-α  hIL-6  hIL-1β | AAGCAGGAGTATGACGAGTCCG  CCTGTAAGTGAAAAGCCATTGGATA  TGTTTGCTGTGAAGACGGTGTC  CAGACAGCCCTAACAAAGCAC  GTCAGATCATCTTCTCGAACC  TCCAGAACAGATTTGAGAGTAGTG  AGATGATAAGCCCACCTCTACAG | GCCTTCATACATCTCAAGTTGG  TCAACAGGGCAGCAAACAAT  GGTGGGAATGGTGGTCACG  CAGAGAGGAGGGTAACCAGTC  CAGATAGATGGGCTCATACC  GCATTTGTGGTTGGGTCAGG  ACATTCAGCACAGGACTCTC |
