# Supplementary material for: BIG1 controls macrophage pro-inflammatory responses through ARF3-mediated PI(4,5)P2 synthesis
Source: Cell Death Dis. 2020 May 15;11(5):374. doi: 10.1038/s41419-020-2590-1 (PMC7229175; doi:10.1038/s41419-020-2590-1)
Supplement: Supplementary file 2 — Supplemental Figure Legends [file 41419_2020_2590_MOESM2_ESM.docx]

**Supplemental Figure Legends**

**Title:** BIG1 controls macrophage pro-inflammatory responses through ARF3-mediated PI(4,5)P2 synthesis

**Running title:** BIG1 regulates TLR4-MyD88 signaling

**Authors**

Authors: Lixin Liu^1#^, Sulin Zhang^1#^, Yirui Wang^1^, Weilian Bao^1^, Yile Zhou^2,3^, Wenzhen Dang^1^, Xu Wang^1^, Haidong Li^1^, Xinyue Cao^1^, Yan You^1^, Hao Fang^2,3^*, Xiaoyan Shen^1^*

**Author affiliation**

1 Department of Pharmacology & the Key Laboratory of Smart Drug Delivery, Ministry of Education, School of Pharmacy, Fudan University, Shanghai, China

2 Department of Anesthesiology, Minhang Branch, Zhongshan Hospital, Fudan University, Shanghai, China

3 Department of Anesthesiology, Zhongshan Hospital Affiliated Fudan University, Shanghai, China

**Note:** # These authors contributed equally to this work.

***Correspondence**

* Xiaoyan Shen, M.D., Ph.D., Department of Pharmacology & the Key Laboratory of Smart Drug Delivery, Ministry of Education, School of Pharmaceutical Sciences, Fudan University, No. 826, Zhangheng Road, Pudong New Area, Shanghai 201203, China. Tel./fax: +86-21-51980182. E-mail address: shxiaoy@fudan.edu.cn.

* Hao Fang, M.D., Ph.D., Department of Anesthesiology, Minhang Branch, Zhongshan Hospital, Fudan University, No. 3071 Hechuan road, Minhang District, Shanghai 201100, China. Tel. +86-21-51876888. E-mail address: [drfanghao@163.com](mailto:drfanghao@163.com).

**Figure S1. Identification results of myelogenous BIG1 conditional knockout mice.**

**A,** Genotype identification of wild-type control (Lyz2-Cre^-^BIG1^fl/fl^) and myelogenous BIG1 conditional knockout (Lyz2-Cre^+^BIG1^fl/fl^) mice. **B,** BIG1 mRNA levels in WT and BIG1^-/-^ BMDMs were measured by RT-qPCR. Mean ± SEM is presented and analyzed by Student’s *t*-test (****P* < 0.001; *n*=3). **C,** Representative immunoblots of BIG1 in WT and BIG1^-/-^ BMDMs.

**Figure S2. BIG1 did not responded to FSL-1 and Pam3csk4 stimulation in BMDMs and THP-1 derived macrophages.**

A, WT BMDMs were separately stimulated with FSL-1 (100 ng/ml) and Pam3csk4 (100 ng/ml) for 12 h, the indicated proteins were analyzed by Western blot. B, WT and BIG1^-/-^BMDMs were stimulated with FSL-1 (100 ng/ml) and Pam3csk4 (100 ng/ml) for 12 h. After treatment, the levels of TNF-α, IL-6 and IL-1β mRNA were measured by RT-qPCR. C, THP-1 cells were treated with FSL-1 (100 ng/ml) and Pam3csk4 (100 ng/ml) for 12 h, after incubating with PMA (100 ng/ml) for 24 h, the indicated protein were analyzed by Western blot. D, THP-1 derived macrophages transfected with negative control siRNA (NC) or BIG1 siRNA (SiRNA) were untreated or treated with FSL-1 (100 ng/ml) and Pam3csk4 (100 ng/ml) for 12 h. Total RNA was extracted. The levels of BIG1, TNF-α, IL-6 and IL-1β mRNA were measured by RT-qPCR.

Data show pooled technical replicates from three independent experiments (panels B and D). All immunoblot data are representative of three independent experiments with similar results.

**Figure S3. Myeloid cell-specific BIG1-cKO protected organ function of sepsis mice from LPS-induced endotoxemic shock.**

WT and myeloid cell-specific BIG1 KO (BIG1 cKO) mice were intra-peritoneally administered with either LPS (50 mg/kg) or isometric saline. **A and B,** Serum ALT and AST levels of WT and BIG1 cKO mice were measured at the indicated time point. Mean ± SEM is presented and analyzed by Student’s t-test (****P* < 0.001; *n*=6). **C,** The mRNA expression levels of WT and BIG1 cKO sepsis mice hepatic proinflammatory cytokines measured by RT-qPCR at the indicated time point. Mean ± SEM is presented and analyzed by Student’s t-test (****P* < 0.001; *n*=6). **D,** The mRNA expression level of the indicated cytokines in lung section from WT and BIG1 cKO sepsis mice were measured by RT-qPCR at the indicated time period. Mean ± SEM is presented and analyzed by Student’s t-test ( ****P* < 0.001; *n*=6).

**Figure S4. Myeloid cell-specific BIG1-cKO mice were protected organ function of sepsis mice in the CLP model.**

WT and BIG1-cKO mice were subject to CLP surgery. **A and B,** Serum ALT and AST levels of WT and BIG1 cKO sepsis mice were measured at the indicated time point. Mean ± SEM is presented and analyzed by Student’s t-test ( ****P* < 0.001; *n*=6). **C,** The mRNA expression levels of WT and BIG1 cKO sepsis mice hepatic proinflammatory cytokines measured by RT-qPCR at the indicated time point. Mean ± SEM is presented and analyzed by Student’s t-test (**P* < 0.05, ***P* < 0.01, ****P* < 0.001; *n*=6). **D,** The mRNA expression level of the indicated cytokines in lung section from WT and BIG1 cKO sepsis mice were measured by RT-qPCR at the indicated time period. Mean ± SEM is presented and analyzed by Student’s t-test (**P* < 0.05, ***P* < 0.01, ****P* < 0.001; *n*=6).

**Figure S5. BIG1 mediates the LPS-stimulated inflammatory response that is independent of ARF1 activation.**

**A,** The interference efficiency of ARF1 in WT BMDMs. Mean ± SEM is presented and analyzed by Student’s *t*-test (****P* < 0.001; *n*=3). **B,** WT BMDMs transfected with si-ARF1 or negative control siRNA were treated with or without LPS (100 ng/ml) for 30 min. Total lysates were subjected to Western blot with indicated antibodies and the relative quantification of proteins were shown. Mean ± SEM is presented and analyzed by Student’s *t*-test (****P* < 0.001; *n*=3). **C,** WT, BIG1^-/-^ BMDMs and BIG1^-/-^ BMDMs infected with active mutant ARF1 (Q71L) or vector were treated with or without LPS (100 ng/ml). After treatment for 30 min, total lysates were subjected to western blot. **D,** WT, BIG1^-/-^ BMDMs and BIG1^-/-^ BMDMs infected with active mutant ARF1 (Q71L) or vector were treated with or without LPS (100 ng/ml). After treatment for 6 h, the mRNA expression levels of TNF-α, IL-6 and IL-1β were measured by RT-qPCR. Mean ± SEM is presented and analyzed by one way ANOVA (**P* < 0.05, ***P* < 0.01, ****P* < 0.001; *n*=5). **E,** THP-1 derived macrophages transfected with negative control siRNA (NC) or ARF1 siRNA (SiRNA) were treated without or with LPS (100 ng/ml) for the indicated time. Total RNA was extracted. The interference efficiency of ARF1 and the expression of TNF-α, IL-6 and IL-1β mRNA were measured by RT-qPCR. Mean ± SEM is presented and analyzed by Student’s *t*-test or one way ANOVA (***P* < 0.01, ****P* < 0.001; *n*=3).

**Figure S6. BIG1 deficiency has no effects on the internalization of TLR4.**

**A and B,** TLR4 expression on cell surface was analyzed by ﬂow cytometry at 0, 15, 30, 60, 120 min after LPS (100 ng/ml) stimulation. TLR4 internalization was quantified. Mean ± SEM is presented and analyzed by Student’s *t*-test (not significant; *n*=3). **C,** WT and BIG1^-/-^ BMDMs were treated with or without Alexa Fluor 488 conjugated LPS (100 ng/ml) for 0, 30, 60 min, the distribution of LPS was visualized by confocal microscope. Scale bars, 20μm. **D,** BMDMs from WT and BIG1-cKO mice were treated with or without bacterial lipoprotein, BLP (250 ng/ml) for 60 min. The cell lysates were subjected to immunoblotting with the indicated antibodies.
