# Supplementary figures and images for: BIG1 controls macrophage pro-inflammatory responses through ARF3-mediated PI(4,5)P2 synthesis
Source: Cell Death Dis. 2020 May 15;11(5):374. doi: 10.1038/s41419-020-2590-1 (PMC7229175; doi:10.1038/s41419-020-2590-1)

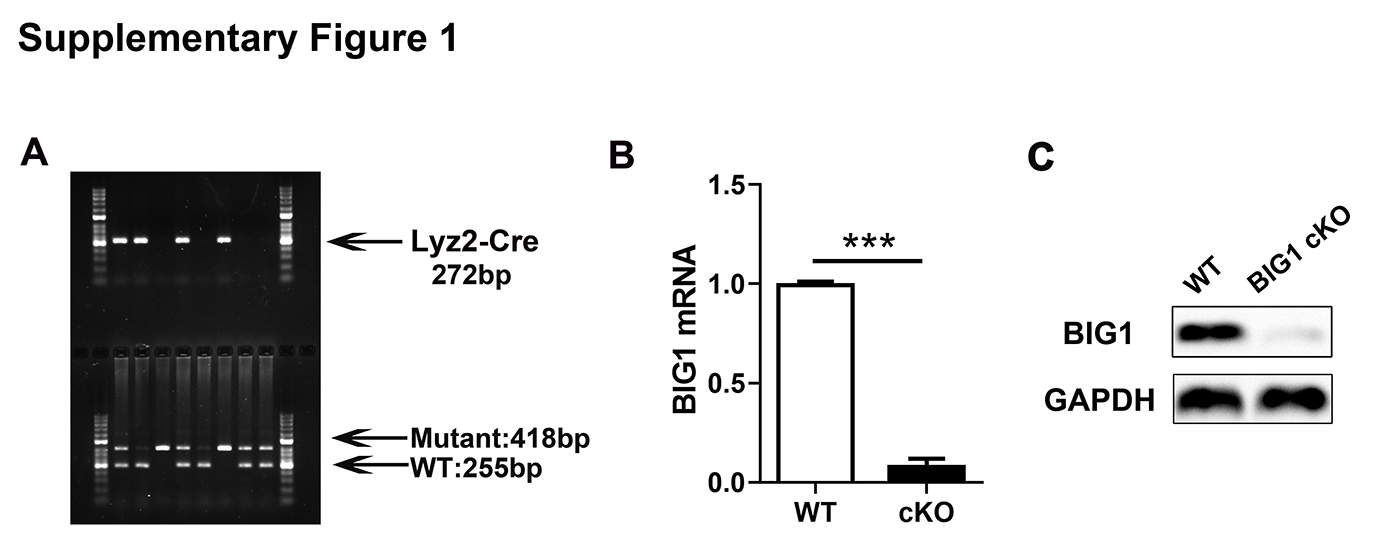

Supplement: Supplementary file 3 — Figure S1 [file 41419_2020_2590_MOESM3_ESM.tif]

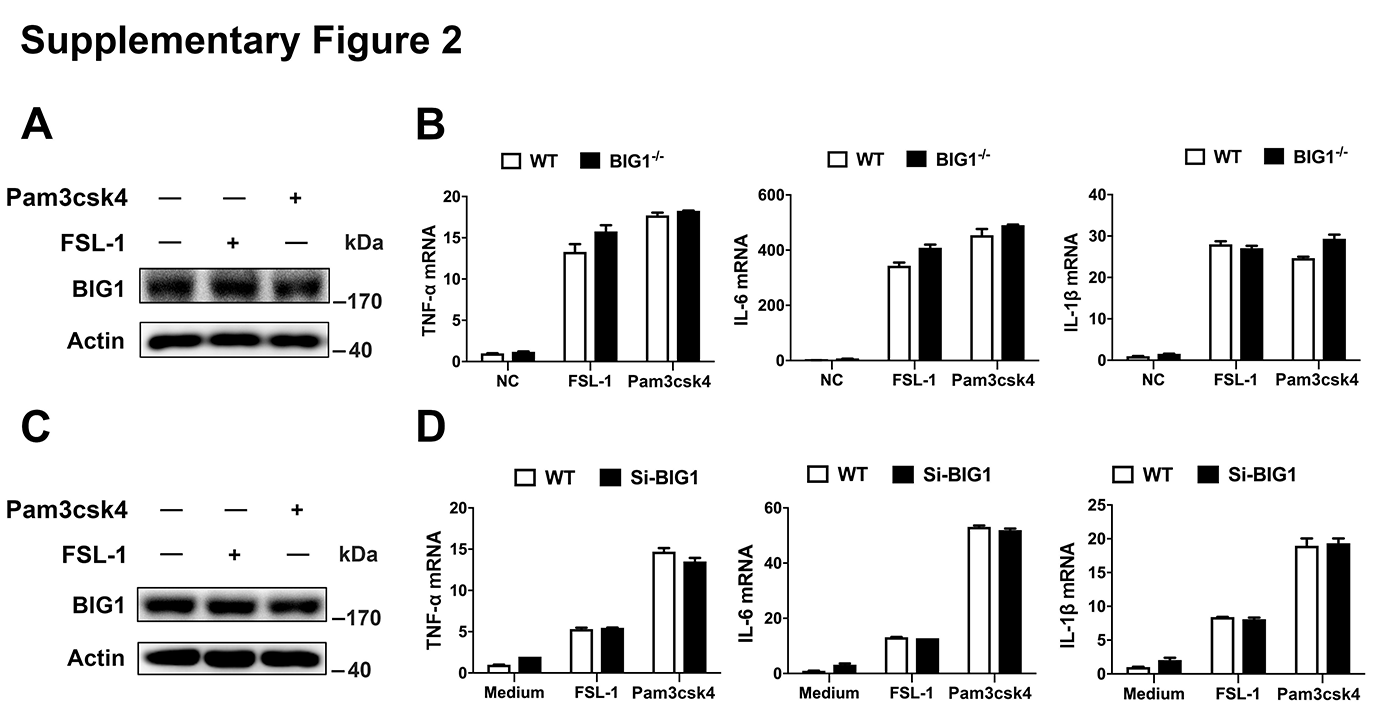

Supplement: Supplementary file 4 — Figure S2 [file 41419_2020_2590_MOESM4_ESM.tif]

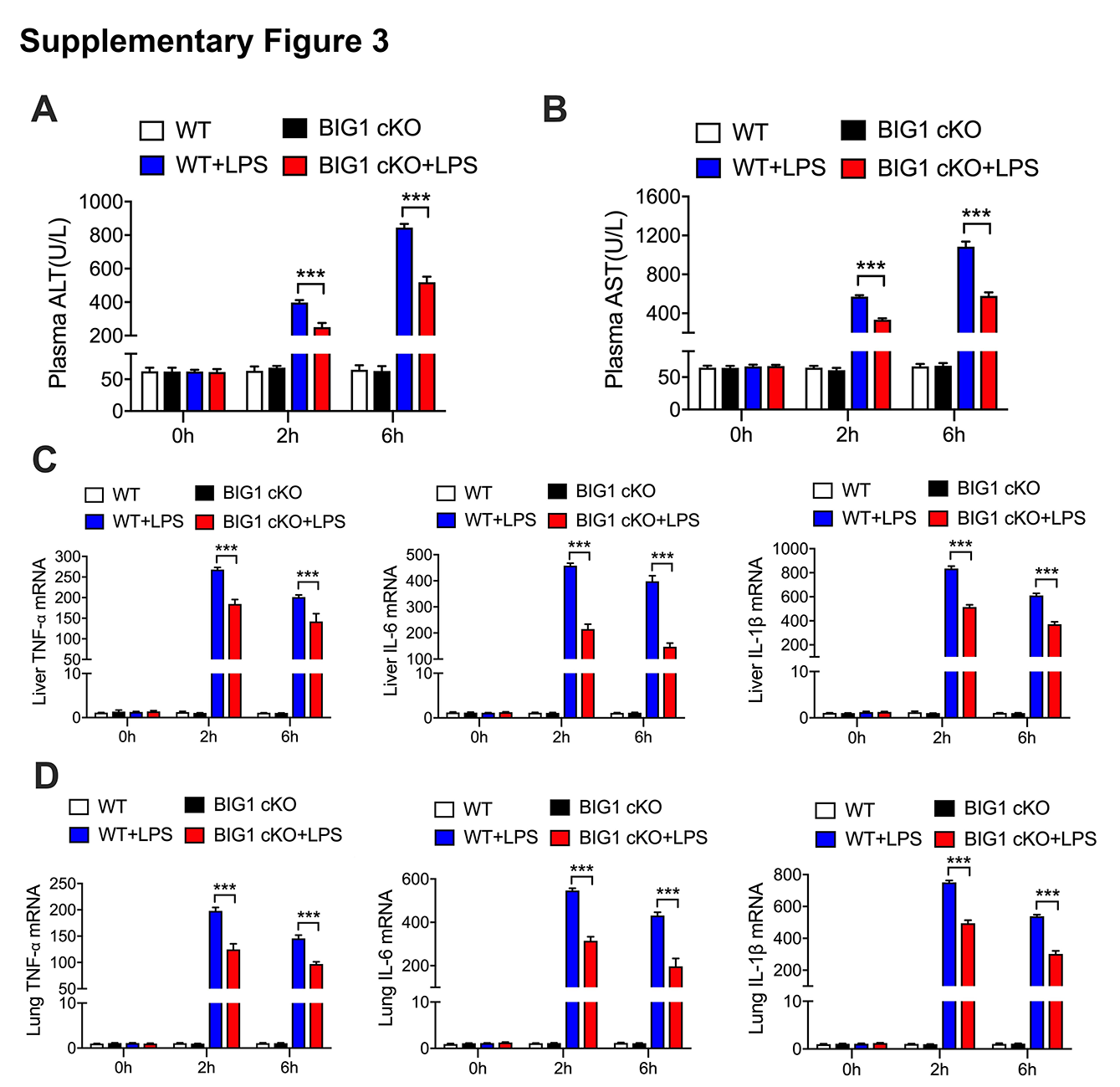

Supplement: Supplementary file 5 — Figure S3 [file 41419_2020_2590_MOESM5_ESM.tif]

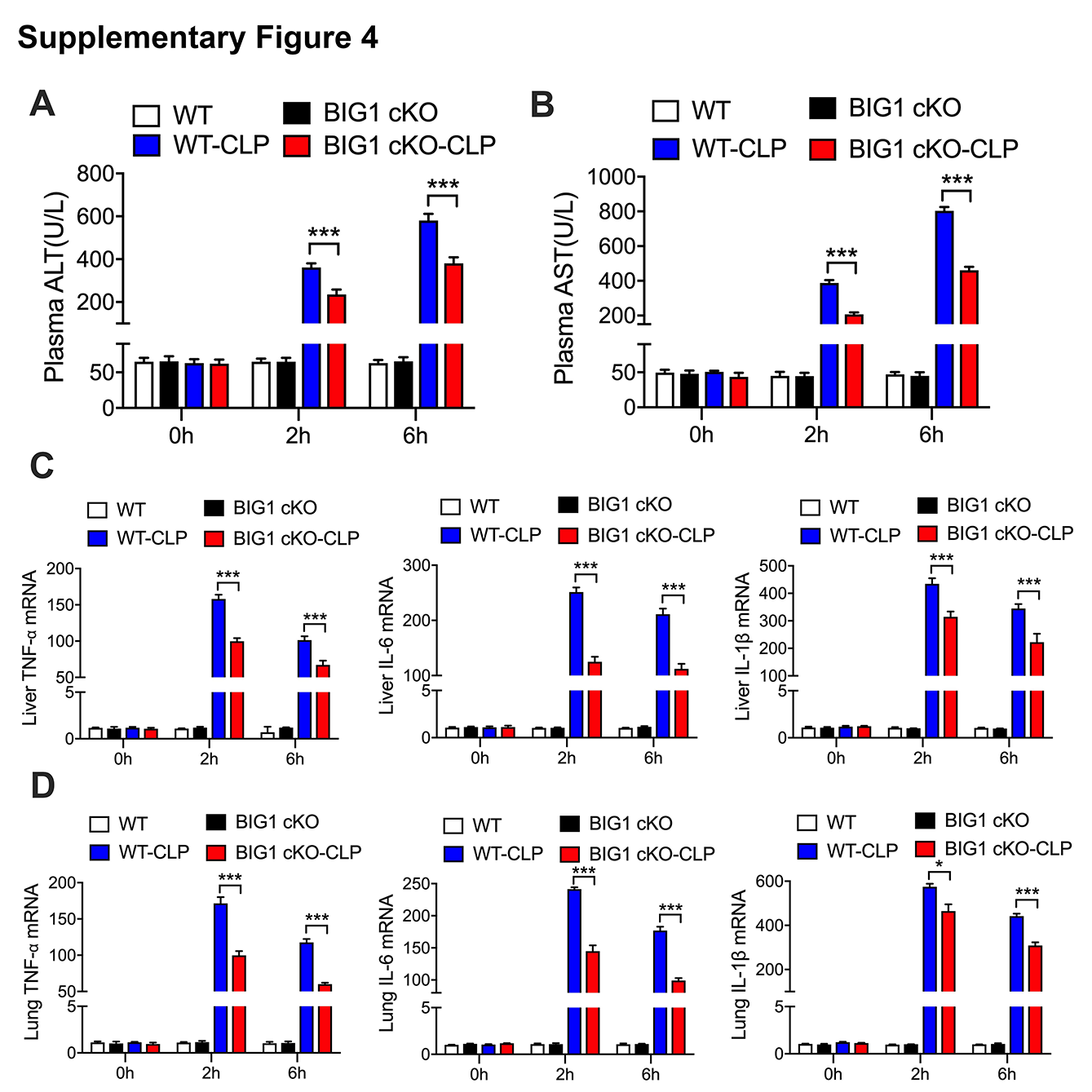

Supplement: Supplementary file 6 — Figure S4 [file 41419_2020_2590_MOESM6_ESM.tif]

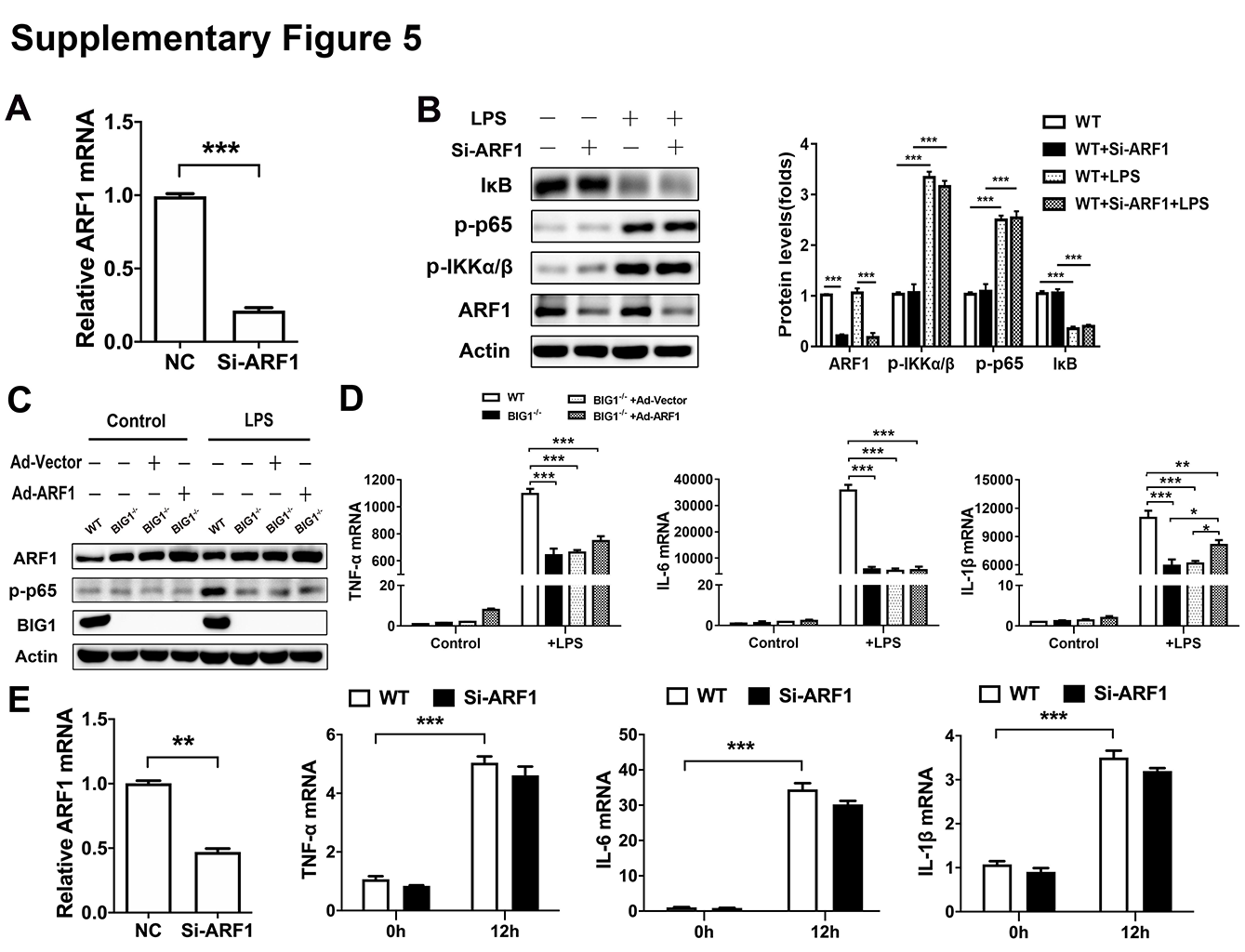

Supplement: Supplementary file 7 — Figure S5 [file 41419_2020_2590_MOESM7_ESM.tif]

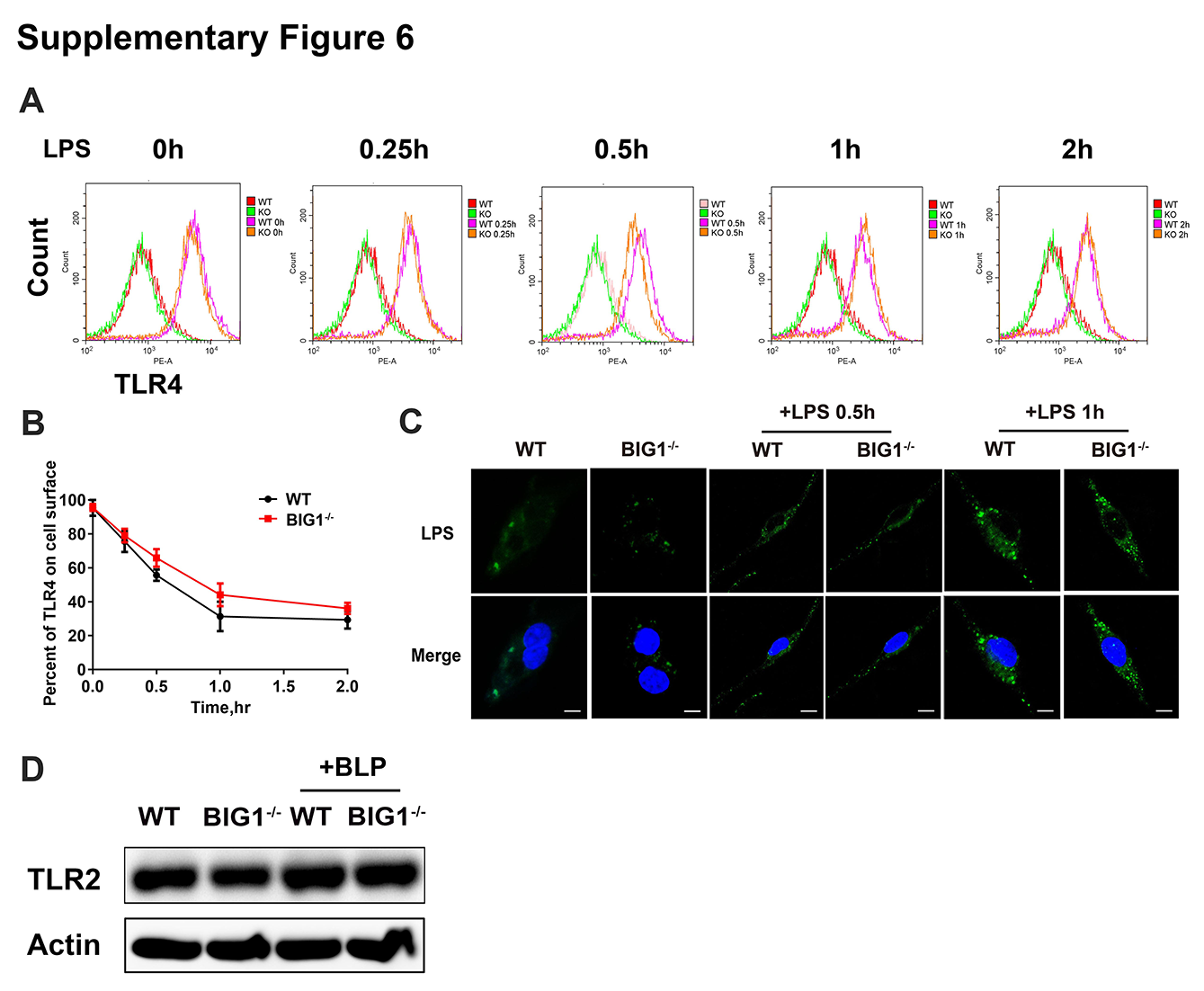

Supplement: Supplementary file 8 — Figure S6 [file 41419_2020_2590_MOESM8_ESM.tif]
